# Small intestinal microbiota composition and the prognosis of infants with ileostomy resulting from distinct primary diseases

**DOI:** 10.1186/s12876-020-01366-0

**Published:** 2020-07-13

**Authors:** Tian Qian, Haitao Zhu, Li Zhu, Chao Chen, Chun Shen, Rong Zhang

**Affiliations:** 1 Department of Neonatology, Children’s Hospital of Fudan University, 399 Wanyuan Rd, Shanghai, 201102 P. R. China; 2 Department of Clinical Nutrition, Children’s Hospital of Fudan University, 399 Wanyuan Rd, Shanghai, 201102 P. R. China; 3 Department of Pediatric Surgery, Children’s Hospital of Fudan University, 399 Wanyuan Rd, Shanghai, 201102 P. R. China

**Keywords:** Ileostomy, Microbiota, Necrotizing enterocolitis, Nutrition, Infant

## Abstract

**Background:**

Studies of microbiota composition of infants with small intestinal ostomy due to various etiologies are limited. Here, we characterized the intestinal microbiota of neonates with ileostomy resulting from distinct primary diseases.

**Methods:**

Fifteen patients with necrotizing enterocolitis, eight patients with meconium peritonitis, and seven patients with Hirschsprung’s disease were included in the study. The small intestinal microbiota composition in infants with ileostomy caused by these diseases was studied.

**Results:**

Microbial diversity in neonatal ileostomy fluid was generally low, and was dominated by members of the Proteobacteria and Firmicutes phyla. At the genus level, the most abundant were *Klebsiella*, *Escherichia-Shigella*, *Streptococcus*, *Clostridium* sensu stricto 1, *Enterococcus*, and *Lactobacillus*. *Streptococcus* and *Veillonella* are related to carbohydrate metabolism and immunity, and breastfeeding can increase the proportion of these potentially beneficial bacteria. The proportion of *Bifidobacterium* in the breastfeeding group was higher than that in the non-breastfeeding group, and incidence of colitis and sepsis was reduced in the breastfeeding group. The proportion of *Bifidobacterium* increased and incidence of colitis and sepsis decreased in the breastfeeding group compared with the non- breastfeeding group, but there was no significant difference. The increase in body weight in the breastfeeding group was observed to be higher than in the non-breastfeeding group.

**Conclusions:**

Excessive *Klebsiella* and *Escherichia-Shigella* and low abundance of *Streptococcus*, *Veillonella* and *Faecalibacterium* suggests that the small intestinal microbiota is in an unhealthy state after ileostomy. However, *Streptococcus*, *Faecalibacterium*, and *Veillonella* species were frequently present, suggesting that expansion of these bacteria might assist the development of the immune system after surgery.

## Background

Enterostomy formation is one of the important surgical treatments for infants with acute abdominal disease. It is commonly performed in response to a variety of intestinal conditions, including necrotizing enterocolitis (NEC), meconium ileus, focal intestinal perforation, intestinal atresia and volvulus. Extensive resections of the small intestine may lead to development of functional short bowel syndrome (SBS). SBS is a malabsorption disorder characterized by fluid and electrolyte imbalances that are consequent to extensive bowel resection. These infants are placed on long-term parenteral nutrition (PN) and enteral nutrition (EN) to compensate for their nutritional deficiencies, which may have a considerable impact on microbiota structure in the small intestine.

The small intestine is the main site for digestion and absorption of nutrients, including almost all proteins, lipids, monosaccharides, and starch [1]. Although the abundance of microbial species in the small intestinal is relatively low, because of rapid peristalsis and secretion of bactericidal substances [2], the small intestinal microbiota has functions that include regulation of immunity, metabolism, and the endocrine system. The impact of these effects on host health can be substantial [3]. Hayashi reported that the majority of microbes in the jejunum and ileum were aerobic bacteria and facultative anaerobes, which included *Streptococcus*, *Lactobacillus*, *Enterococcus*, and γ-*Pseudomonas* [4]*.* Wang et al. reported that *Streptococcus* accounted for more than 60% of the jejunal microbiota, and *Clostridium* clusters IVa and IV were the dominant species in the distal ileum [5]. It has been shown that *Streptococcus*, *Veillonella*, and *Lactobacillus* are the main microorganisms in the small intestine and that they are involved in intestinal immune regulation [6]. Therefore, alterations of small intestinal microbiota composition might affect disease symptoms. Due to the location of the small intestine, its microbiota is difficult to sample. As a result, only a few studies have focused on small intestinal microbiota.

Multiple primary diseases can lead to ileostomy, including Hirschsprung’s disease (HD), meconium peritonitis (MP), and NEC. During infancy, the composition of the intestinal microbiota is relatively simple and can be influenced by a variety of factors. In this study, we characterized the intestinal microbiota of infants with an ileostomy resulting from defined primary diseases. The association between clinical symptoms, intestinal microbiota composition and therapeutic effects were also comprehensively analyzed. This study provides valuable data for informing postoperative care and future clinical practice.

## Methods

### Study subjects and sample collection

We recruited 30 infants with small intestinal ostomy caused by various primary diseases. All patients were recruited from the Children’s Hospital of Fudan University, as shown in Tables 1 and S1. All patients were categorized into the following three groups according to their primary diseases: HD (8 cases), MP (7 cases), and NEC (15 cases). After the infants had reached full enteral feeding (daily enteral feeding > 120 ml/kg), samples of ileostomy fluid were collected and stored at − 80 °C until microbiota analysis. The infants received oral formula or breast milk, and were not fed any solid food. This study was approved by the Human Investigation Committee of the Children’s Hospital of Fudan University. Written informed consent was obtained from all parents.

**Table 1:** Clinical information for patients with different primary diseases (data were presented as median)

Infant characteristic	All patients with ileostomy (*n* = 30)	Primary diagnosis		
		Hirschsprung’s disease (HD, *n* = 8, H01-H08)	Meconium peritonitis(MP, *n* = 7, M01-M07)	Necrotizing enterocolitis (NEC, *n* = 15, N01-N15)
Male sex, n	20	4	3	13
Gestational age (week)	34 (25–40)	36.8 (30–40)	36.3 (34–40)	31.4 (25–39)
The operation day age (day)	19.8 (0–77)	24.1 (1–50)	4.7 (0–17)	24.5 (3–77)
Operative weight (kg, range)	2.2 (1.0–3.5)	2.6 (1.3–3.5)	2.7 (1.6–3.3)	1.7 (1.0–3.5)
Remaining small bowel length (cm)	86.9 (40–160)	96.9 (80–110)	79.8 (40–160)	84.8 (72–100)
Breast feeding, n	8	1	1	6
Parenteral nutrition duration (day)	38.3 (11–91)	33.5 (11–91)	48.3 (17–85)	36.1 (20–74)
Colitis, n	12	6	3	3
Septicemia, n	22	7	4	11
Shannon index	1.17 (0.2–1.83)	1.37 (0.78–1.8)	1.14 (1.01–1.55)	1.07 (0.2–1.83)

### 16S rRNA gene sequencing

Bacterial genomic DNA was extracted from the ileostomy fluid samples using the E.Z.N.A. Stool DNA Isolation Kit (Omega Bio-Tek, Inc., GA, USA). The 16S rDNA V3-V4 region was amplified by PCR using barcoded Illumina adapter-containing primers 341F (5′-CCTACGGGNGGCWGCAG-3′) and 805R (5′-GACTACHVGGGTATCTAATCC-3′) [7]. The final 16S rRNA gene amplicon library was sequenced on the MiSeq platform (Illumina) using a 2 × 300 bp paired-end protocol. Illumina MiSeq sequencing was performed by Shanghai Mobio Biomedical Technology Co., Ltd. (China). Raw sequencing data have been submitted to the NCBI Sequence Read Archive under accession number PRJNA553095.

Clean data was extracted from raw data using USEARCH 8.0 with the following criteria: (i) sequences of each sample were extracted using each index with zero mismatch, (ii) sequences with overlaps of less than 50 bp and with error rate of the overlap greater than 0.1 were discarded, (iii) sequences less than 400 bp after merging were also discarded. Quality-filtered sequences were step-wise clustered into operational taxonomic units (OTUs) at a similarity of 97% using UPARSE (version 7.1 http://drive5.com/uparse/) [8]. The phylogenetic affiliation of each 16S rRNA gene sequence was analyzed by RDP Classifier (http://rdp.cme.msu.edu/) against the Silva (SSU123)16S rRNA database using a confidence threshold of 70% [9].

### Statistical analyses

Estimates of alpha diversity were calculated with standard methods using QIIME 1.9.0 [10]. The structure and characteristics of the microbial communities after each treatment step were analyzed using R. Hierarchical clusters at the genus level were generated with the Bray-Curtis average distance method using R. Student’s t-test was performed to analyze normally distributed data. Chi-square test or Fisher’s exact test was used for analysis of categorical variables. The built in function p.adjust in R was used (method = “BH”) to control the false discovery rate. Genera with relative abundances higher than 1% in at least one of the samples were included. A canonical correspondence analysis (CCA), which describes the relationships between the corresponding environmental parameters and relatively abundant genera in microbial communities, was performed using Canoco 4.5.

## Results

### Low microbial diversity and chaotic microbial succession

**Fig. 1:**
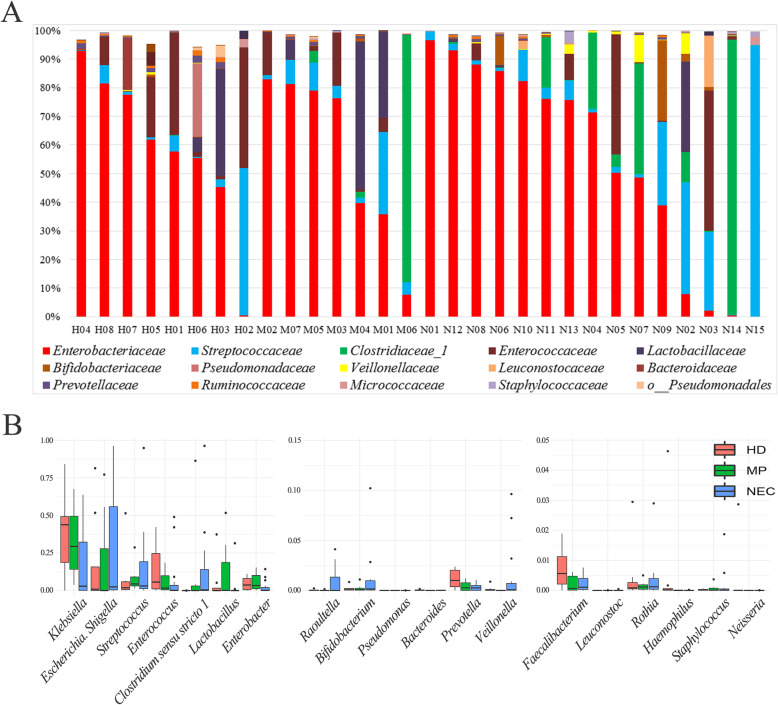
Microbial communities in infants with ileostomy. Panel **a** shows the proportion of family within the microbiota in patients with HD (H01-H08), MP (M01-M07), and NEC (N01-N15). Panel **b** shows the proportion of genus within the microbiota in patients with HD (H01-H08), MP (M01-M07), and NEC (N01-N15). HD: Hirschsprung’s disease; MP: meconium peritonitis; NEC: necrotizing enterocolitis

To understand the relationship between the distinct primary diseases and shifts in intestinal microbiota composition, the patients were classified into three groups according to their primary disease; namely, the HD, MP, and NEC groups. There were 8, 7, and 15 patients in the HD, MP, and NEC groups, respectively (Table 1). Shannon diversity index was relatively low in infants with ileostomy as shown in Tables 1, S1 and Figure S1. Clinical profiles of patients are summarized in Table S1. Proteobacteria and Firmicutes were the most prevalent phyla, followed by Actinobacteria and Bacteroidetes (Figure S1). These four phyla accounted for more than 99.9% of the microbial communities in all samples. In these patient samples, Enterobacteriaceae (56.4%) was the dominant taxonomic family, followed by Streptococcaceae (11.8%), Clostridiaceae 1 (9.6%), Enterococcaceae (8.7%), Lactobacillaceae (5.5%), and Bifidobacteriaceae (1.6%). These six most common taxonomic families accounted for more than 93.6% of the microbial communities in all samples (Fig. 1a).

### Structural differences in intestinal microbiota from individuals

**Fig. 2:**
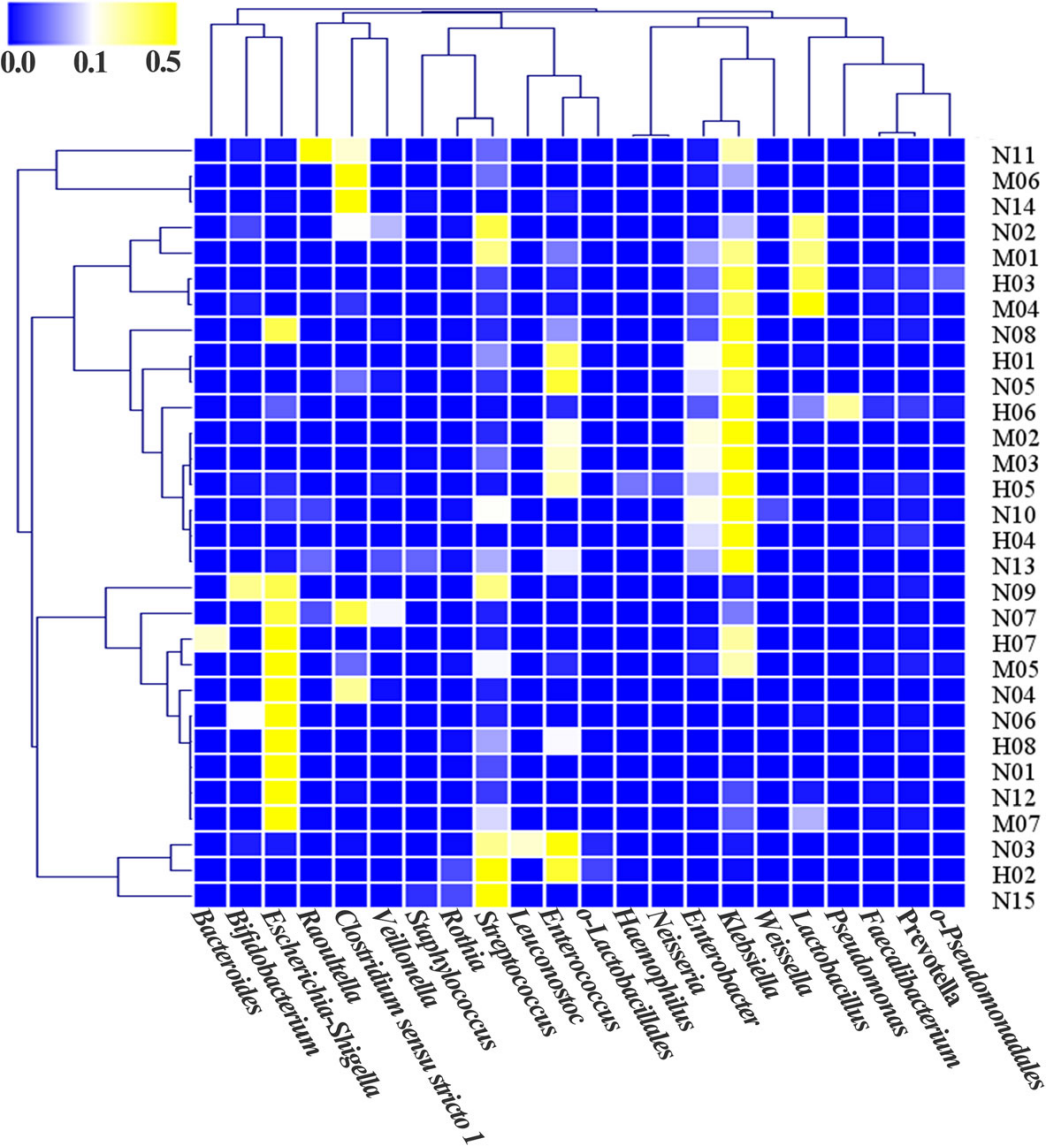
Heatmap of the relative abundance of the signature genera in infants with ileostomy. Each parallel lane corresponds to one sample. Relative abundance percentage of each genus in the corresponding whole community is indicated by the color scale. Blue: 0, white: 0.01 (1%), and yellow: 0.5 (50%)

To further explore microbial composition of the three groups, core genera were examined in detail (Fig. 1b and S2). One hundred and nineteen OTUs were obtained from all the samples. The most abundant bacteria were *Klebsiella* (26.0% on average), followed by *Escherichia–Shigella* (24.6%), *Streptococcus* (11.8%), *Clostridium* sensu stricto *1* (9.6%), *Enterococcus* (8.7%), and *Lactobacillus* (5.5%). Cluster analysis demonstrated that the relative abundance of *Klebsiella* was higher in some patients (Fig. 2), and *Klebsiella* and *Enterobacter* always appeared simultaneously (indistinguishable based on the 16S V4 region). Cluster analysis of intestinal microbiota composition in patients was also performed at the level of genera (Fig. 2). Microbial community structures were not well grouped according to the different etiologies. Although different patients have diverse small intestinal microbiota profiles, *Escherichia*-*Shigella* and *Klebsiella* are commonly detected and are relatively high (Figs. 2 and S2). In addition, *Clostridium* sensu stricto 1 was the main species in samples M06 and N14, and *Raoultella* was the main species in sample N11 (Fig. 2). The microbiota composition analysis of ileostomy fluid thus reveals that individual subjects have distinct microbiota structures. However, despite these distinct characteristics, the microbiotas contained common species. For example, *Streptococcus* was detected in almost every sample, albeit with variable relative abundance (Figs. 2 and S2).

### Bifidobacterium and breastfeeding

**Fig. 3:**
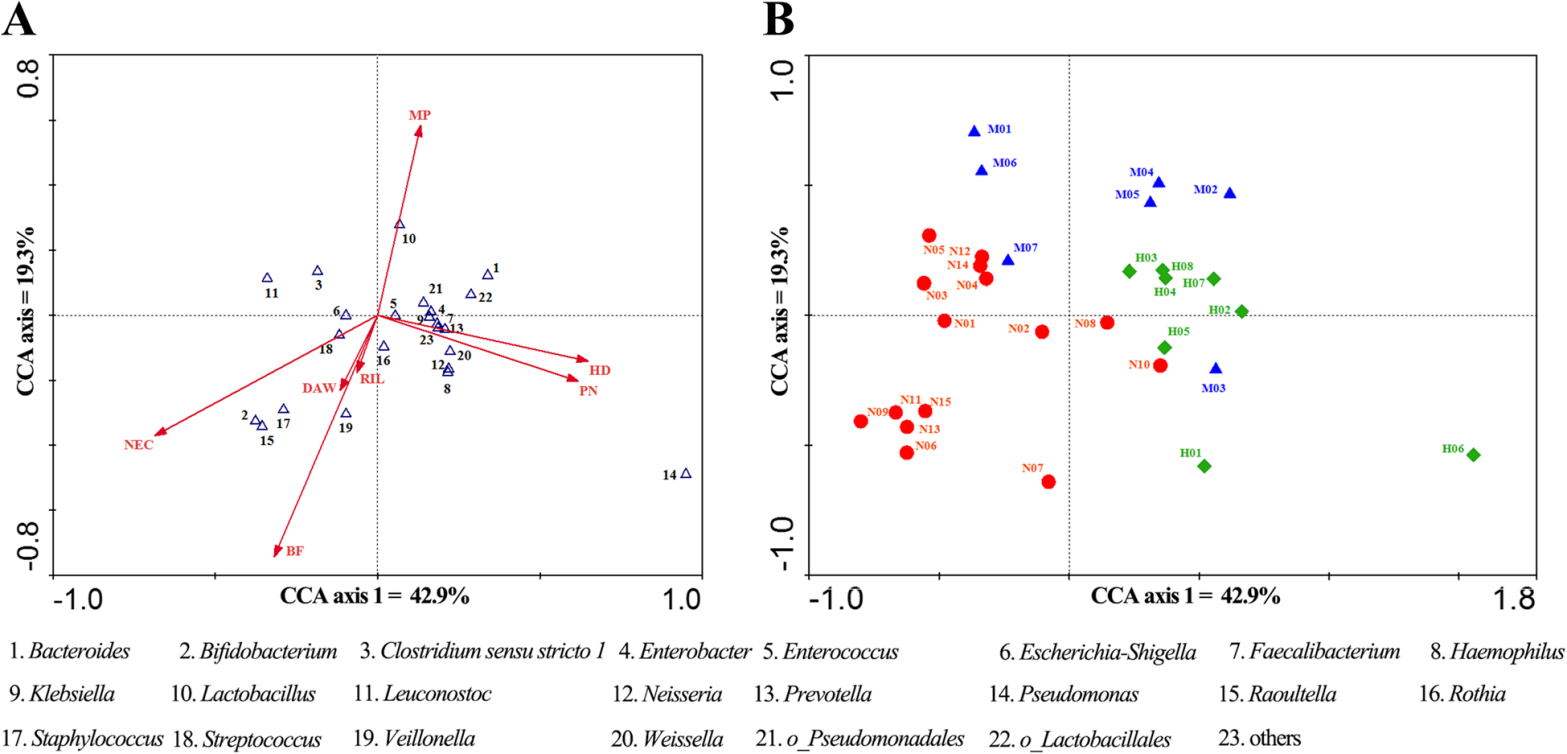
Canonical correspondence analysis (Canoco 4.5 CCA) of the effects of different factors. **a** Ordination diagram of canonical correspondence analysis exhibits bacterial species and environmental variables and primary diseases (arrows). **b** Influence of environmental variables and primary diseases on samples, which plotted on the same axes as the first panel. Axis 1 explains 42.9% of the variation, whereas axis 2 explains an additional 19.3%. HD: Hirschsprung’s disease; MP: meconium peritonitis; NEC: necrotizing enterocolitis; DAW: duration of antibiotic withdrawal, BF: breastfeeding; PN: parenteral nutrition; RIL: residual intestinal length

Similarly to *Faecalibacterium*, *Bifidobacterium* was also detectable in some samples at low abundance. Eight of the 30 patients were breast-fed (Table S1). *Streptococcus* is associated with carbohydrate metabolism, and breastfeeding can increase the proportion of this beneficial bacterium in the small intestine. Breast milk usually contains *Bifidobacterium*, *Lactobacillus*, and *Streptococcus*, and another probiotic species *Staphylococcus* is often found on areola skin; therefore, these bacteria can be transferred directly from mother to baby during breastfeeding [11, 12]. We found that the incidence of colitis and sepsis was lower in the breast-fed group than in the non-breast-fed group, while the weight gain was higher in the breast-fed group than in the non-breast-fed group, but there was no significant difference (Table 2). CCA analysis revealed that *Bifidobacteria*, *Streptococci*, and *Veillonella* were closely associated with breastfeeding (Fig. 3).

**Table 2:** Influence of breastfeeding on ileostomy patients

	No BF (22/30)	BF (8/30)	*p*-value
Growth rate of body mass (g/d)	21.2	27.4	0.091 ^#^
Colitis (n)	11	1	0.152^※^
Sepsis (n)	18	4	0.202^※^
*Bifidobacterium*(%)	0.34	4.90	0.180^#^
*Streptococcus*(%)	9.30	18.40	0.429^#^

^#^ T-Test; ^※^ Chi-square test

### Other influences on intestinal microbiota composition

To investigate potential effects of other external factors on intestinal microbiota structure, we performed CCA analysis. As shown in Fig. 3, more than 60% of the variability in microbiota structure was explained by the CCA axes. According to our data, different etiologies have a significant influence on the microbial community structure. Taking account of external factors, we grouped infants with ileostomy into three different primary diseases (Fig. 3). In addition to the etiologies, the intestinal microbiotas were influenced by several factors, which included: duration of antibiotic withdrawal, breastfeeding, PN duration, and residual intestine length.

## Discussion

The intestinal microbiota represents an important factor affecting human health, exhibiting evolutionarily conserved roles in host metabolism, immunity, development, and behavior [13, 14]. It has been reported that the fecal microbiota of babies born by caesarean section is rich in *Enterobacter*, *Streptococcus australis*, and *Veillonella*, which are mainly derived from the skin, oral cavity, and the surrounding environment during birth. The intestinal microbiota of neonates born by vaginal delivery is rich in *Escherichia-Shigella*, *Bacteroides*, and *Bifidobacterium*, with *Escherichia-Shigella* being the most abundant genus [15]. It has been reported that enrichment of genes involved in vitamin K2 synthesis in newborns is associated with a high abundance of *Escherichia-Shigella,* an organism with the ability to synthesize vitamin K2 [15, 16].

*Streptococcus* and *Veillonella* often coexist in the intestinal ecosystem [17, 18], and might cooperate in the metabolic process, so their combined immunoregulatory properties have also been investigated [6]. Since the source of these bacteria in newborns is maternal, it can be implied that they play a role in clinical recovery and immune development of ileostomy patients after surgery. Data suggest that microorganisms in the small intestine are mainly involved in simple carbohydrate metabolism, a task predominantly performed by specific species including *Streptococcus* and *Veillonella*. Our results are consistent with findings of previous studies; that is, enterobacteria always include *Streptococcus* and *Veillonella* spp. in the ecosystem, and these are the most active members of the ileal microbiota [3]. Functions of *Streptococcus* and *Veillonella* are often complementary, however, in our study we found a lower abundance of *Veillonella* in patients. *Streptococci* can metabolize a variety of carbohydrates, while *Veillonella* can utilize lactic acid as a carbon and energy source, and are proposed to metabolize lactic acid produced by *Streptococcus* [19, 20]. Metatranscriptomic analysis of ileostomy fluid also revealed that the presence of *Streptococcus* in the small intestine could be related to the transportation and metabolism of carbohydrate substrates [17]. *Escherichia* or other microbes can substitute for this role when the abundance of *Streptococcus* is insufficient [17]. Further studies identifying underlying mechanisms are needed to explain the contribution of *Streptococcus* and *Veillonella* to immunity and homeostasis in pediatric patients after ileostomy, and this could help guide future clinical practice. *Faecalibacterium*, a prominent member of the Firmicutes phylum, has been reported to possess immunoregulatory and anti-inflammatory properties [21]. For example, *Faecalibacterium prausnitzii* produces anti-inflammatory proteins, and Crohn’s disease (CD), a disorder associated with disrupted microbial ecology, is characterized by decreased abundance of *F. prausnitzii*. Moreover, the decrease in *F. prausnitzii* is associated with an increased risk of symptom recurrence after ileal CD surgery [21, 22]. A limitation of our study is that the sample size is too small to link specific bacterial populations to the 3 primary diseases. Grouping microbial community structure according to different etiologies was not effective. In contrast, different patients have diverse small intestinal microbiota profiles, and dominated by Escherich*ia-Shigella* and *Klebsiella*. In addition, many of the clinical parameters we described could have influenced the dataset, including duration of antibiotic removal, breastfeeding, PN duration, and residual intestine length.

Nutritional factors have a significant influence on the composition of the intestinal microbiota*.* Breast milk is an optimal source of nutrition for infants, providing proteins, carbohydrates, lipids, fats, and some micronutrients essential for infant growth. Breast milk also contains several biologically active components that include immunoglobulins and oligosaccharides, and resulting microbiota plays a critical role in infant intestinal homeostasis and immune development [23]. Our study found a trend of increased growth rate of body mass and decreased prevalence of colitis and sepsis in breastfeeding patients compared to non-breastfeeding patients. Thus, breastfeeding is likely to play a role in maintaining the intestinal function and improving postoperative recovery of such patients.

Excessive *Klebsiella* and *Escherichia-Shigella,* and low abundance of *Streptococcus*, *Veillonella*, and *Faecalibacterium,* indicated that the small intestinal microbiota of our donors was in an unhealthy state. It has been reported that the relative abundance of *Enterobacter* or *Klebsiella* is significantly higher in breast milk of mothers of infants infected with rotavirus as well as in the intestine of the infected neonates, whereas the abundance of *Streptococcus* and *Staphylococcus* was significantly lower [24]. Our data suggest a correlation between the presence of *Klebsiella/Enterobacter* and neonatal gastrointestinal disease, as well as a potential protective effect of *Staphylococcus* or *Streptococcus*. A frequent occurrence of *Klebsiella* species has also been reported in pathogenic genera identified in SBS II patients [25]. Therefore, it would be beneficial to study further the effects of *Klebsiella/Enterobacter* in ileostomy and its prognosis, in order to provide novel hypotheses that guide future clinical practice.

## Conclusions

This is the first report to define characteristics of the intestinal microbiota of neonates with ileostomy resulting from distinct etiologies. The results reveal that patients with small intestinal ostomy subsequent to different etiologies have dissimilar microbial community characteristics. In addition, we observed relatively individualized small intestinal microbiota profiles. The diversity of species in the small intestinal fluid of neonates with ostomy was low, reflected by low Shannon indexes. Bacteria were mainly distributed within the phyla Proteobacteria and Firmicutes, and less frequent within Actinobacteria and Bacteroidetes. More research is warranted to understand the relationship between intestinal dysbiosis and primary disease, and the associated influences of microbiota on the prognosis of infants with ileostomy.

## Supplementary information

**Additional file 1.**

**Additional file 2.**

## Data Availability

The datasets used and analysed in the current study are available from the corresponding author upon reasonable request.

## Abbreviations

NEC: Necrotizing enterocolitis
SBS: Short bowel syndrome
PN: Parenteral nutrition
EN: Enteral nutrition
HD: Hirschsprung’s disease
MP: Meconium peritonitis
CCA: Canonical correspondence analysis
CD: Crohn’s disease
