# Supplementary material for: Small intestinal microbiota composition and the prognosis of infants with ileostomy resulting from distinct primary diseases
Source: BMC Gastroenterol. 2020 Jul 13;20:224. doi: 10.1186/s12876-020-01366-0 (PMC7359560; doi:10.1186/s12876-020-01366-0)
Supplement: Supplementary file 2 — Additional file 2. [file 12876_2020_1366_MOESM2_ESM.docx]

Supplementary Material

## Supplementary Figures


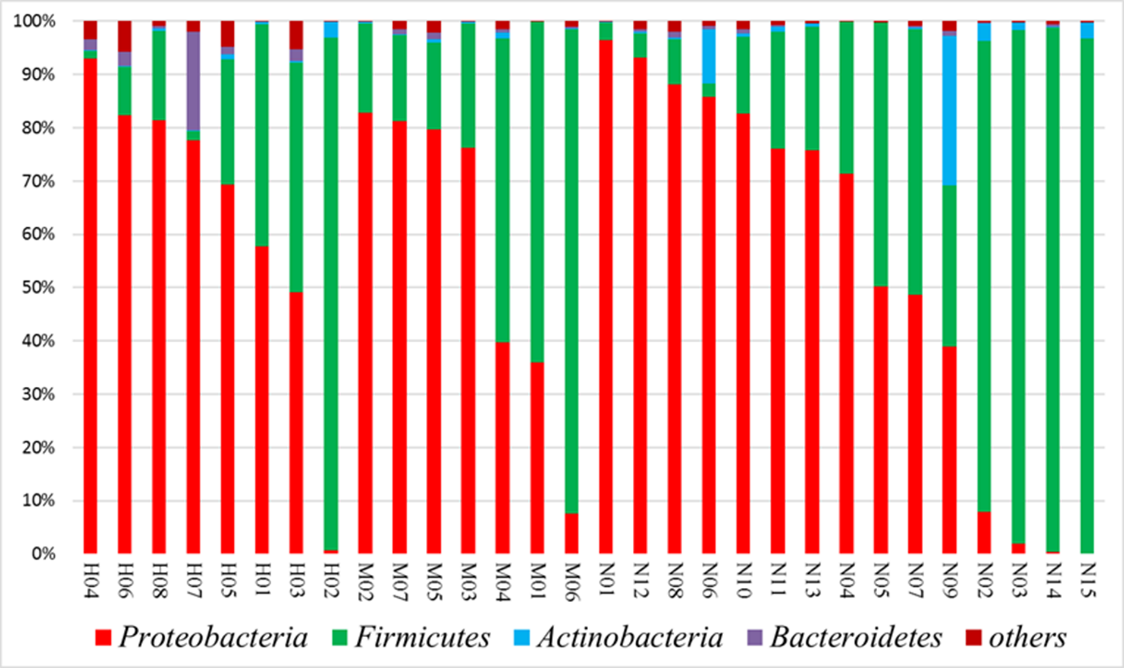


A

B

**Supplementary Figure 1.** A, Microbial communities in infants with ileostomy. The figure shows the relative abundance of the four dominant taxonomic phyla in patients with HD (H01-H08), MP (M01-M07), and NEC (N01-N15). HD: Hirschsprung's disease; MP: meconium peritonitis; NEC: necrotizing enterocolitis. B, Shannon index of all samples.


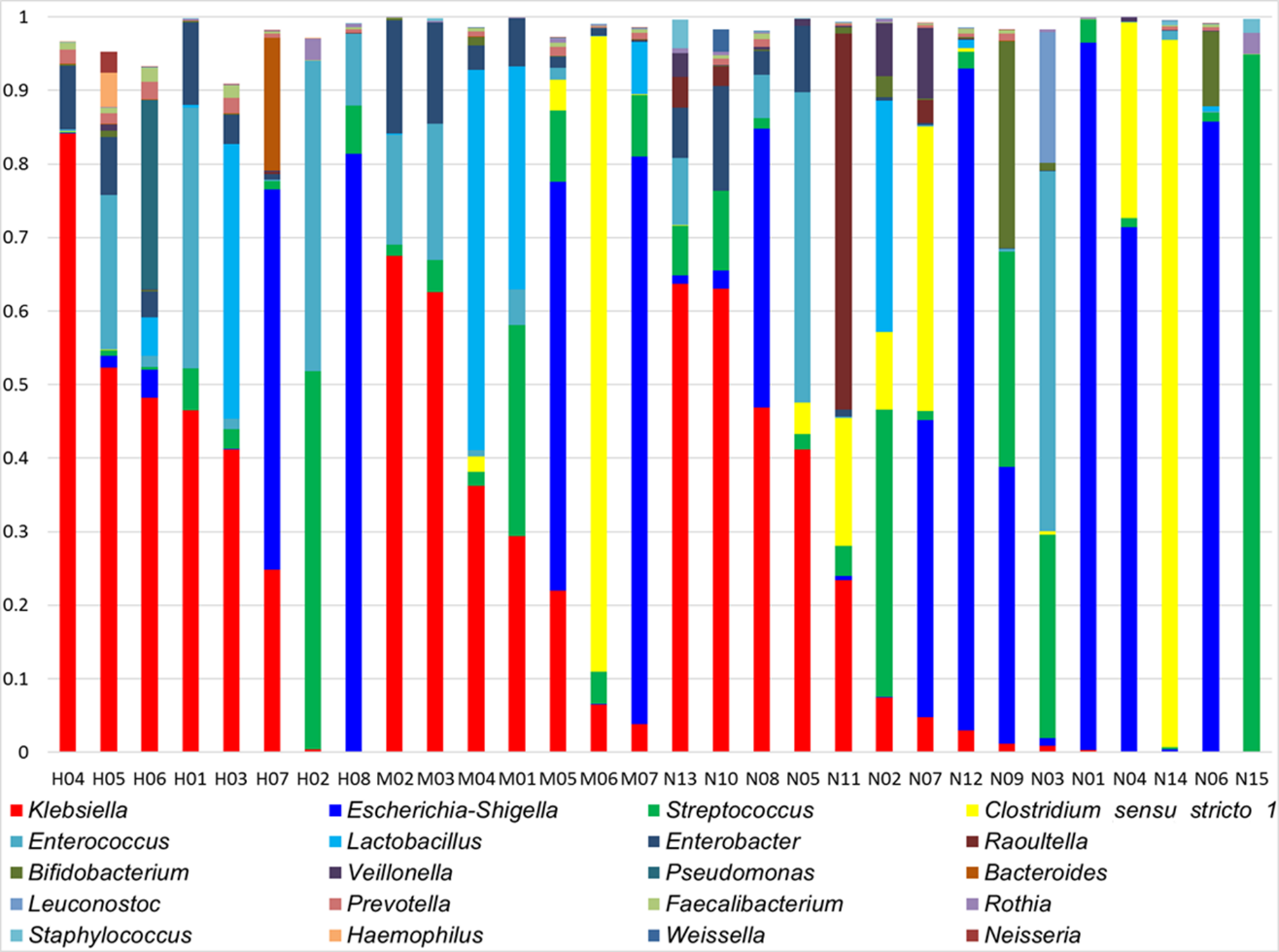


**Supplementary Figure 2.** Microbial communities in infants with ileostomy. The figure shows the relative abundance of the 20 taxonomic genera in patients with HD (H01-H08), MP (M01-M07), and NEC (N01-N15). HD: Hirschsprung's disease; MP: meconium peritonitis; NEC: necrotizing enterocolitis.
